# Iodine-125 brachytherapy for brain tumours - a review

**DOI:** 10.1186/1748-717X-7-30

**Published:** 2012-03-06

**Authors:** Silke B Schwarz, Niklas Thon, Katharina Nikolajek, Maximilian Niyazi, Joerg-Christian Tonn, Claus Belka, Friedrich-Wilhelm Kreth

**Affiliations:** 1 Department of Radiation Oncology, Ludwig-Maximilians-University Hospital, Marchioninistr. 15, 81377 Munich, Germany; 2 Department of Neurosurgery, Ludwig-Maximilians-University Hospital, Marchioninistr. 15, 81377 Munich, Germany

**Keywords:** Stereotactic brachytherapy, interstitial brachytherapy, seed, iodine-125, brain tumour

## Abstract

Iodine-125 brachytherapy has been applied to brain tumours since 1979. Even though the physical and biological characteristics make these implants particularly attractive for minimal invasive treatment, the place for stereotactic brachytherapy is still poorly defined.

An extensive review of the literature has been performed, especially concerning indications, results and complications. Iodine-125 seeds have been implanted in astrocytomas I-III, glioblastomas, metastases and several other tumour entities. Outcome data given in the literature are summarized. Complications are rare in carefully selected patients.

All in all, for highly selected patients with newly diagnosed or recurrent primary or metastatic tumours, this method provides encouraging survival rates with relatively low complication rates and a good quality of life.

## Introduction

The implantation of radioactive material into tumours was proposed by Pierre Curie in 1901 [1]. In 1951, Friedlander and Orr prepared iodine-125 by alpha bombardment of natural antimony [2]. In 1965, this nuclide was finally introduced in interstitial cancer therapy (prostate, lung cancer and lymph nodes) [3]. Mundinger played a pioneering role in the development of the concept of brachytherapy in brain tumours: he began to implant iridium-192 wires into gliomas at 1960; since 1979 he has preferred iodine-125 seeds [4,5]. Nowadays, temporary implantation of iodine-125 seeds is preferred for interstitial brachytherapy in brain tumours. Despite favourable physical and biological characteristics of brachytherapy and well established implantation techniques, so far brachytherapy treatment of brain tumours has been performed in only a few centres worldwide. The current review considers radiobiological characteristics, indications, and treatment effects of iodine-125 brachytherapy (including side effects) for patients with brain tumours.

## Review

### Rationale for stereotactic iodine-125 brachytherapy

Interstitial brachytherapy aims for a highly localized devitalisation of a well defined treatment volume, thereby avoiding damage of the surrounding non-neoplastic tissue. Treatment volume and target volume are ideally identical. The minimal-invasive, spatially precise stereotactic implantation technique, in combination with favourable physical characteristics of the radioactive sources, enable the accurate application of highly focused necrotizing tissue dose with a steep fall-off from the centre to the periphery. The pronounced dose inhomogeneity within the target volumes make iodine-125 brachytherapy an attractive concept, particularly for selected patients suffering from small, circumscribed tumours at any location of the brain. Of note, stereotactic brachytherapy must not be confused with stereotactic radiotherapy, which exhibits much less dose inhomogeneity and lower intratumoural (non-necrotizing) doses, and stereotactic radiosurgery, which is characterized by the absence of any effects of fractionation [6-10].

### Physical characteristics, dosage and dosimetry

Conventional fractionated irradiation is administered with a dose rate of about 200cGy/min. In contrast, interstitial irradiation aims for much lower dose rates of < 100cGy/h. This continuous low-dose rate irradiation increases the therapeutic ratio as the ongoing repair of sublethal irradiation doses is more effective in non-neoplastic tissue, than in tumour (see below).

Iodine-125 emits gamma-rays with a very low average photon energy of 28.5 keV [11,12], has a specific gamma-ray constant of 1.32-1.45R· cm^2^/mCi·h [11], a long half-life of 59.4-60.2 days [2,12-14], a half-value tissue thickness of 2 cm and for lead of 0.025 mm, which enables easy shielding [4,15]. Iodine-125 implants generate a typically extreme dose inhomogeneity within target volumes that ranges from highly necrotizing doses surrounding the seeds to the ultimate form of fractionation at the periphery of target volumes [16] (Table 1).

**Table 1:** Isotopes used for brachytherapy in brain tumours (Adapted from [16]).

Isotope	Emission	Mean energy (MeV)	Half-life (days)
Iodine-125	γ	0.028	60

Iridium-192	γ	0.38	74

Phosphorus-32	β	0.69	14

Rhenium-186	β	0.36	4

Yttrium-90	β	0.93	3

There are two forms of iodine-125 brachytherapy techniques: temporary and permanent [17] (Table 2). Currently, temporary implants are preferred as permanent implants bear an increased risk of prolonged oedema [18-20]. Exclusively low activity iodine-125 seeds (< 20mCi) are preferred for slowly proliferating processes, such as low-grade gliomas, to achieve a dose of 50-60Gy at the tumour margin; the dose rate is usually extremely low (10cGy/h; range 5-20cGy/h). High-dose-rate brachytherapy (> 30cGy/h), as being achieved with high activity seeds of up to 40mCi, however, may be required for rapidly-proliferating high-grade gliomas [12,21,22]. Of note, high dose rates (30-60cGy/h) alone or in combination with external beam radiation have been shown to be associated with a high frequency of radiogenic complications (30-50%) [23-25].

**Table 2:** Parameters of permanent and temporary iodine-125 seeds (Adapted from [13,147]).

	Permanent seeds	Temporary seeds
**Activity per source**	0.1-3mCi	3-50mCi

**Dose**	80-700Gy	30-200Gy

**Dose rate**	3-30cGy/h	3-125cGy/h

**Number of sources per case**	1-171	1-28

**Time to dose**	infinity	4-50 days

To calculate the total dose for permanent iodine-125 implants the mean life time of 87 days is used [26]. As compared with alternative permanent implants (Au-198 and Pd-103), I-125 has been shown to be more effective on slow growing tumours (tumour doubling time of > 10 days) [27].

Principles of seed positioning aim for a good coverage of the target volumes with an acceptable uniformity of the dose distribution by a minimal number of catheter implants while sparing the surrounding anatomical structures (i.e. normal brain tissue, vessels, optic nerve etc.) from radiation exposure [28-33]. Usually, one to five seeds, encapsulated in the tip of a Teflon catheter, are necessary to achieve a conformal interstitial irradiation, even of complexly shaped tumour volumes. Of note, iodine-125 seeds are available in various shapes, making their dose distribution non-isotropic, which must be considered for therapy planning [11,14,34-40]. The tri-planar treatment-planning system is based on stereotactic computed tomography (CT) and magnetic resonance imaging (MRI), as well as novel metabolic imaging data. It allows positioning of seeds within target volumes, generate and display the resulting isodose distribution and calculate approach angles [32,41,42]. Care has to be taken that the high dose zones (> 150Gy) always lie within the tumour tissue and that vessels are not adjacent to these zones [43].

The definition of target volumes has changed over time. In the beginning of seed implantation in brain tumours, the target volume was mostly determined to be the tumour visible in CT, plus an isotropic margin of 0.5 cm [23,30,44-47]. Presently, image fusion with magnetic resonance imaging and positron emission tomography allows a better visualisation and more precise definition of the target volume [16]. Schupak et al. found out that the technical accuracy had an impact on the local failure rate but not on the overall survival of the patients [48].

The following parameters should be documented for each stereotactic brachytherapy: prescribed total dose, dose rate, minimum tumour dose, percentage of the tumour receiving less than the prescribed dose, maximum dose in "normal" brain at 1 cm from the tumour border, number of seeds, activity of seeds, volumes of tumour and surrounding tissue irradiated to various total doses and dose rates [49].

In summary, the accurate dose distribution, the rapid dose decrease from the centre of the treatment volume towards the periphery, and the high doses in the tumour and the low doses in the normal tissue, are great physical advantages of iodine-125 implants for brain tumour therapy [16].

### Biological characteristics

#### Theoretical models and cell culture experiments

Dale et al. have adapted the linear-quadratic (LQ) model for protracted radiotherapy, incorporated tumour repopulation and tumour shrinkage factors, to allow radiobiological assessment of permanently implanted iodine-125 seeds [50-52]. Using the conventional linear-quadratic model with an α/β = 10Gy for tumour tissue and an α/β = 3Gy for healthy tissue and given a repopulation resulting in a loss of 0.4Gy/d, 60Gy of temporary implants in 5 days are almost equivalent to 100Gy permanent implant dose [53].

Even though the conventional LQ model describes well the radiobiological effects of an implant at the boundary of the target volume, it does not account for the effects of extreme dose inhomogeneity associated with brachytherapy [54]. Characteristic tissue effects associated with the high dose zone in the vicinity of the implanted source (≥ 200Gy) have been described experimentally, i. e. the development of circumscribed radionecrosis with temporary changes in capillary permeability with a sometimes extensive oedema and concomitantly reduced regional cerebral blood flow [18-20,55,56] (see below).

The relative biological effectiveness (RBE) of iodine-125 compared to Cobalt-60 is 1.3 to 1.5 [57-59]. Compared to iridium-192, iodine-125 has a RBE of 1 [60].

The overall low-dose-rate of iodine-125 allows reoxygenation for increasing radiosensitivity and cancels out the repair of sublethal damage. Normal tissue irradiation is essentially reduced because the exposure level decreases by the inverse square of the distance from the source. Therefore, the rate of tumour cell killing of brachytherapy has been thought to be much greater than that of conventionally fractionated teletherapy [22].

#### Animal experiments and histological studies

Iodine-125 brachytherapy reduces histological features that are prognostic for tumour progression, i.e. cellularity, pleomorphism, vessel hyperplasia and degree of mitosis, and lowers the proliferating cell nuclear antigen, a marker for late G1- and S-phases of the cell cycle [61].

To examine how the tumour microenvironment (perfusion, oxygen partial pressure) changes in response to low-dose-rate brachytherapy, experiments with a mouse hypoxic tumour model have been performed. The perfusion is elevated at a distance of 2-4 mm from the seed, starting three days after implantation, and pO_2 _is increased one to three days after implantation. Therefore, additional external beam radiotherapy should be most advantageous 1-2 days after implantation, when pO2 is high, and chemotherapy most advantageous 3-4 days after implantation, when perfusion is high [62].

Autopsy material showed that the early phase after implantation is characterized by migrating macrophages and removal of necrotic debris. In the established phase, a necrotic centre and a reactive zone around have been found. The reactive zone consists of a narrow inner rim of microglial accumulation and a broader outer area with astrocytic gliosis, vascular proliferation, activated microglia and infiltration by macrophages. In the burned-out phase the necrosis undergoes liquefaction, the microglia rim is replaced by end-stage macrophages and the reactive zone is transformed into astrocytic gliosis equivalent to a scar [55,63].

A radiomorphological triple ring formation has been identified to be characteristic after seed implantation (see below) and correlates with histopathological alterations including a coagulative necrosis in the central radiolucent region, a fibrinoid necrosis of vessel walls within the ring enhancement and an adjacent spongiosis and gliosis, as well as oedema [18]. With increasing time after implantation the ring enhancement moves outward [64].

The effect of permanent iodine-125 implants on the blood-brain barrier (BBB) function was additionally studied in normal canine brains. The breakdown of the BBB occurs as soon as 7 days after implantation. The radiation lesion enlarges rapidly from day 7 to 72, then increases more slowly until day 392 and is relatively decreased in size in 2 years. Furthermore, the finding of the three zones with calcified necrosis, a narrow fluid-filled zone and a narrow rim of viable but damaged tissue in which there is breakdown of the BBB for up to 1 year before returning to an almost normal function at 2 years, were confirmed. Increased permeability was most pronounced in white matter as compared to grey matter [19].

#### Summary of biological characteristics

Due to the continuous low-dose rate irradiation, especially in the periphery of the target volume and at the edge to normal tissue, sublethal damage can be repaired and long term side effects of the late-responding tissue can be avoided. In the centre of the treated volume, highly focused necrotizing intratumoural doses with a steep dose decrease from the centre to the periphery, such as in radiosurgery, can be achieved. Therefore, seeds combine the advantages of fractionated radiotherapy (repair of surrounding normal tissue) and radiosurgery (tumour cell death irrespective of radiosensitivity) in one modality.

Because repopulation and redistribution during the treatment are of minor importance in the therapy of low grade gliomas, low dose rate seed implantation appears to be a rational therapeutic strategy [16].

### Stereotactic biopsy and implantation techniques

Obtaining tissue diagnosis and molecular genetic profiling are mandatory for each suspected brain tumour. This can be routinely performed by means of stereotactic biopsy procedures that integrate CT, MRI and metabolic imaging data for trajectory planning and enable minimal-invasive collection of representative specimens throughout entire tumour volumes (including biologically "hot spots" if present), especially for those tumours that are not eligible for gross open tumour resections [16,65,66]. Therefore, the authors promote a stage concept of interstitial brachytherapy after versatile tissue diagnosis and molecular genetic profiling of the brain tumours have been obtained.

**Figure 1:**
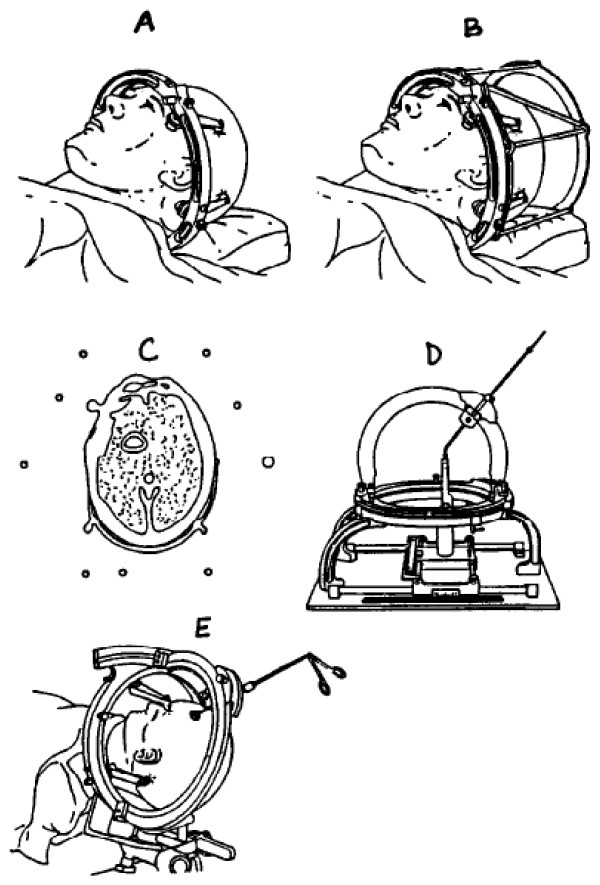
**Sequence of steps in using the BRW stereotactic system **[21]. A: The head ring is fixed to the skull. B: The localizing ring with its nine carbon-fibre rods is attached to the head ring for CT scanning. C: A sample CT cut shows the circular cross sections of the localizing rods. D: The pin and arc device are attached to the phantom base plate to check the computed settings for the desired trocar placement. E: The pin and arc device are attached to the head ring on the patient.

Seed implantation procedure is a multidisciplinary treatment involving neurosurgeons, radiation oncologists, neuro-radiologists and physicists [16]. A detailed description of the stereotactic implantation technique has been given in several reports by Kreth et al. [16,67,68]. Seed implantation is usually performed under general anaesthesia. After attaching a stereotactic ring to the patient's head, CT scans with a localizing system mounted on the base ring are obtained. Then the computer based treatment planning process starts, including 3D image reconstruction, image fusion with magnetic resonance imaging and/or positron emission tomography, finding the optimum seed localisations and trajectories based on the coordinate system of the frame. The implantation of the iodine-125 seeds (length: 4.5 mm) loaded Teflon catheters is performed via a 2 mm burr-hole for each catheter. CT-scan follow-up is performed one day after surgery and fused with the pre-operative localized CT to control the seed positions. A hospital stay of about three days is required for the implantation procedure. The level of radiation upon discharge is checked and documented by the physicist [12,13,16,17,30,69-74] (Figure 1). Steroids should be administered routinely on the day of implantation and for three days postoperatively at a daily decreasing dose of 24, 12, 8 and 4 mg dexamethasone, respectively. For temporary implants, the skin is re-opened for seed-removal after 20-30 days under local anaesthesia (no stereotactical equipment needed).

### Indications for iodine-125 brachytherapy

Iodine-125 brachytherapy is a valuable treatment option for patients with non-resectable, small, and circumscribed untreated tumours in any location of the brain, as well as local circumscribed recurrences after previously performed percutaneous radiotherapy and/or surgery [75]. Ideally, the diameter of the tumour should be smaller than 3 cm. In case of larger tumour volumes, microsurgery (partial resection) might be combined with brachytherapy [17]. Of note, interstitial brachytherapy can be performed even in cases in which tolerance of the healthy brain tissue has been reached because of previous external irradiation and/or stereotactic radiosurgery [4,76]. Implantation is also a valuable technique in certain paediatric patients [77,78]. For a long time, infratentorial masses, the hypothalamic region, the middle-inferior mesencephalon, the diencephalon, the pons, the corpus callosum and tumours with subependymal or transcallosal spread, involvement of the cerebellum, the brainstem or the deep thalamic regions, involvement of basal ganglia structures, sylvian or interhemispheric fissures, have been considered contraindicated sites for implantation. Furthermore, large tumours > 6 cm or with a tumour volume of > 120 ml, multifocal masses, and those with diffuse margins, patients with a Karnofsky performance status (KPS) of < 60 and significant oedema, have been thought to be not suitable for implantation [12,13,46,73,79-82]. Currently, technical indications have been extended [83,84]. Only diffuse tumours, tumours with a diameter larger than 4 cm, or with infiltration of the corpus callosum, are still considered to be insuitable for implantation [16].

### Follow-up characteristics

During MRI follow-up evaluation, a characteristic triple ring formation can be identified, which should not be confused with tumour progression [64]. The inner zone of this formation refers to the necrotized tumour, the intermediate contrast enhanced zone to a small rim of a still viable tumour, exhibiting increased capillary permeability, and the outer zone to the treatment induced oedema. The ring shaped area of contrast-enhancement is visible 4.5 months to 3 years after implantation [85]. A study on volumetric changes in tumour necrosis, reactive zone and oedema over the time after temporary seed implantation (50-60Gy) has revealed the following: compared to the reference dose volume the necrosis has been 19.9% after 6 months, 8.3% after 14 months and 7.0% after 24 months, the reactive zone 30.4%, 34.8% and 16.7%, respectively, and the oedema 293.2%, 220.5% and 107.0%, respectively. Necrosis has been found in regions with > 79.2Gy with a maximum 6 months after irradiation, a shrinkage between 9 and 15 months and a steady-state after 16 months. The reactive zone has had its maximum 2 months after irradiation, after 12 months the shrinkage has been more pronounced. The maximum of the oedema has been reached after 6 months [86].

Other visible late radiation effects (> 2 months) are decreased enhancement of the tumour region, decreased mass effect, atrophic changes in surrounding brain (widened sulci), calcification in areas immediately adjacent to the seeds, and focal necrosis corresponding to the treatment volume, surrounded by a zone of decreased cerebral metabolism in positron emission tomography (PET) [47].

To monitor iodine-125 brachytherapy effects in low-grade gliomas with PET L-methyl-carbon-11-methionine uptake seems to be more suitable than 2-fluoro-18-1-deoxy-D-glucose (FDG), as it shows a dose dependent decline in uptake [87,88].

A magnetic resonance imaging study compared longitudinally features of patients either treated with external beam radiotherapy alone or in combination with brachytherapy. In both groups, nodular enhancement adjacent to or remote from the resection cavity strongly suggests tumour recurrence. Linear rim enhancement, later on progressing to feathery enhancement, immediately adjacent to the cavity, has been seen in brachytherapy patients only and strongly indicates radiation necrosis [89].

### Clinical indications and results

The results concerning survival achieved with permanent and temporary brachytherapy implants in patients with various brain tumours are summarized in Table 3. For recurrent tumours, survival is measured starting from time of implantation. In the following section indications, clinical outcome, risk limits and side effects are given for different brain tumour entities.

**Table 3:** Results concerning survival achieved with permanent and temporary iodine-125 brachytherapy in patients with various brain tumours.

Study	Number of patients & type of iodine-125 seeds	Dose, dose rate, activity	Diagnoses (number of patients) & additional percutaneous radiotherapy (pRT)	6 months-survival rate	9 months-survival rate	12 months-survival rate	18 months-survival rate	2 year-survival rate	3 year-survival rate	4 year-survival rate	5 year-survival rate	8 year-survival rate	10 year-survival rate	15 year-survival rate	Median survival
Mundinger 1980 [4]	221 iodine-125 & iridium-192		Astrocytomas I (55)						72%		52%				
			Astrocytomas II (76)						69%		44%				
			Astrocytomas III (34)						66%		47%				
			Oligodendrogliomas II/III (26)						65%		27%				
			Glioblastomas (17)												
			Germinomas (13)					19%	30%						

Leibel 1984 [194]	43 temporary	80-120Gy	Recurrent primary tumours preirradiated												> 18 months

Wara 1985 [189]	temporary		Recurrent malignant gliomas												18 months

Leibel 1985 [195]	43 temporary	80-120Gy	Recurrent primary tumours preirradiated												18 months

Leibel 1986 [111]	77 temporary	50-120Gy	Recurrent tumours:												
			Astrocytomas III (42)					49%							22 months
			Glioblastomas (35) all preirradiated					26%							17 months

Gutin 1987 [110]	41 temporary	57.4-120Gy	Recurrent tumours:												
		25-100cGy/h	Astrocytomas III (23)												35 months
		30-40mCi	Glioblastomas (18) preirradiated (44-70.5Gy)												8 months

Wright 1987 [47]	14 temporary	100-280Gy	New tumours (12)												2-32 months
	2 permanent	15-30cGy/h 6-144mCi	Additional pRT (45Gy)												
			Recurrent tumours (4) Preirradiated												
			Glioblastomas (12)												

Willis 1988 [46]	17 temporary	New:	New glioblastomas (5)			100%	75%	25%							
		60Gy	Additional pRT (50.4Gy)												
		35-64cGy/h 34-239mCi													
		Recurrent:	Recurrent tumours (12)	93%		60%	50%	38%							
		80Gy	Astrocytomas III (8)												
		30-65cGy/h 122-320mCi	Glioblastomas (4) preirradiated (50.4-66Gy)												

Leibel 1988 [44]	95 temporary	50-120Gy													
			Recurrent tumours:				55%		40%						20 months
			Astrocytomas III (50)				30%		15%						12 months
			Glioblastomas (45) preirradiated												

Prados 1989 [158]	14 temporary	50cGy/h 10-40mCi	Metastases preirradiated												18 months

Etou 1989 [93]	56 temporary & permanent	90-100Gy	Paediatric tumours:												
			Astrocytomas I (36)			86%			58%		22%				
			Astrocytomas II (14)			79%			36%		21%				
			Astrocytomas III (6)			33%			17%		17%				

Malkin 1989 [127]	21 temporary	60Gy	Astrocytomas III (2)												
		36cGy/h	Glioblastomas (19)												6 months
		79-495mCi	Recurrent tumours (10): Preirradiated												

Yakar 1989 [159]	50 temporary	55-60Gy	New tumours:												11 months (mean)
		40cGy/h	Astrocytomas III (3)												
		4-299mCi	Glioblastomas (17)												
			Additional pRT (50-60Gy)												
			Recurrent tumours:												13 months
			Astrocytomas III (5)												
			Glioblastomas (12)												
			Preirradiated												
			Metastases (13)												8 months (mean)

Leibel 1989 [45]	95 temporary	52.7-150Gy	Recurrent tumours:												
		20-90cGy/h	Astrocytomas III (50)				46%		28%						19 months
		27-372mCi	Glioblastomas (45) preirradiated (40-72Gy)				22%		8%						12 months

Larson 1990 [81]	128 temporary	46-120Gy	New tumours:												
		25-100cGy/h	Astrocytomas III (20)												51 months
			Glioblastomas (13)												22 months
			Additional pRT (60Gy)												
			Recurrent tumours:												
			Astrocytomas III (50)				45%		28%						19 months
			Glioblastomas (45) Preirradiated				22%		8%						12 months

Bernstein 1990 [107]	46 temporary	New:	New tumours:												
		60Gy	Astrocytomas III (23)												14 months
			Additional pRT (50Gy)												
		Recurrent:	Recurrent tumours:												
		70Gy	Astrocytomas III (18)												10 months
		21.8-125cGy/h 93-599mCi	Metastases (3)												
			Radiation-induced tumours (2) preirradiated												

Loeffler 1990 [134]	35 temporary	38-55Gy	Glioblastomas (35)			87%		57%							
		30-60cGy/h 20-50mCi	Additional pRT (59.4Gy)												

Gutin 1990 [109]	55 temporary	50Gy	Astrocytomas III (25)												36 months
			Glioblastomas (30) Additional pRT (60Gy)												20 months

Gutin 1991 [23]	63 temporary	50-60Gy	Astrocytomas III (29)												36 months
		40-60cGy/h 10-40mCi	Glioblastomas (34) Additional pRT (60Gy)												20 months

Clark 1991 [108]	25 temporary	60Gy	Astrocytomas III (4)												30 months
			Glioblastomas (21) Additional pRT												21 months

Loeffler 1991 [135]	25 temporary	39-64Gy	Recurrent Glioblastomas (25)			42%	30%	15%							10 months

Scharfen 1991 [113]	334 temporary	37-150Gy	New tumours:												
		25-100cGy/h	Astrocytomas III (80)					74%							
			Glioblastomas (117)					33%							
			Additional pRT												
			Recurrent tumours:												
			Astrocytomas III (70)					41%							
			Glioblastomas (67)					23%							

Zamorano 1992 [160]	64 temporary	Temporary:	New tumours:			75%		47%							15 months
	50 permanent	60Gy	Astrocytomas III												
		40cGy/h	Glioblastomas												
			Additional pRT (50Gy)												
		Permanent:	pRT→temporary (25)			59%	30%								14 months
		81-143Gy	permanent→pRT (19)			79%		59%							
		4-7cGy/h	pRT→permanent (9)			100%	50%								13 months
			permanent only (9)					100%							
															
			Recurrent tumours:			38%	13%								10 months
			temporary (23)			39%	10%								10 months
			permanent (11)			36%	36%								9 months
			Metastases:												
			temporary (16)			44%	28%								10 months
			permanent (2)			44%	25%								10 months

Zamorano 1992 [161]	50 temporary	55-60Gy	New tumours:												11 months (mean)
															
		40cGy/h	Astrocytomas III (3)												
		4-299mCi	Glioblastomas (17)												
			Additional pRT (50-60Gy)												13 months
			Recurrent tumours:												
			Astrocytomas III (5)												
			Glioblastomas (12)												
			Preirradiated												
			Metastases (13)												8 months (mean)

Sneed 1992 [145]	206 temporary	New:	New tumours:												
		42-76Gy	Astrocytomas III (29)												36 months
			Glioblastomas (34)												20 months
			Additional pRT (60Gy)												
															
		Recurrent:	Recurrent tumours:												
		53-150Gy	Astrocytomas III (50)												19 months
		20-90cGy/h	Glioblastomas (45)												12 months
			Preirradiated												
															
		Hyperthermia:	Recurrent tumours:												
		33-63Gy	Astrocytomas III (16)			81%	65%								
			Glioblastomas (25)				45%								11 months
			Metastases (7) Plus hyperthermia												10 months

Yakar 1992 [115]	62 temporary	Temporary:	New tumours:				40%								14 months
															
		60Gy	Astrocytomas III (7)				43%								13 months
		40cGy/h	Glioblastomas (18)				39%								15 months
			Additional pRT												
			Recurrent tumours:				23%								11 months
			Astrocytomas III (8)				37%								9 months
			Glioblastomas (13)				8%								11 months
			Metastases (16)				25%								10 months
	55 permanent	Permanent:	New tumours:				73%								37 months
		100Gy	Astrocytomas III (36)				79%								37 months
		5cGy/h	Glioblastomas (5)				27%								14 months
			Recurrent tumours:				42%								14 months
			Astrocytomas III (7)				86%								23 months
			Glioblastomas (4)				0%								6 months
			Metastases (3)				33%								4 months

Scharfen 1992 [25]	307 temporary	37-120Gy	New tumours:												
		19-140cGy/h	Non-glioblastomas (68):												
		38-453mCi	Astrocytomas I/II			94%			73%						52 months
			Astrocytomas III			85%			47%						33 months
			Glioblastomas (106)			86%			22%						20 months
			Additional pRT (60Gy)												
			Recurrent tumours:												
			Non-glioblastomas (67):												
			Astrocytomas I/II			68%			30%						19 months
			Astrocytomas III			49%			24%						12 months
			Glioblastomas (66)			50%			15%						11 months

Prados 1992 [112]	88 temporary	50-60Gy	Astrocytomas III (32)						32%						37 months
		40-60cGy/h 10-40mCi	Glioblastomas (56) Additional pRT (40-77Gy)					29%	14%						20 months

Ostertag 1992 [95]	194 temporary 345 permanent	30-70Gy	Astrocytomas I (106)								77%				
		10cGy/h	Astrocytomas II (251)								65%	64%			
			Oligoastrocytomas (44)								80%				
			Oligodendrogliomas (29)								58%				
			Astrocytomas III (75)			49%		36%							8 months
			Glioblastomas (34)			48%		16%							6 months

Kreth 1993 [162]	81 temporary	60Gy	New metastases:												
		10cGy/h	WBRT (40Gy) + seed (38)												17 months
			Seed only (22)												12 months
			WBRT (40Gy) only (49)												8 months
			Recurrent metastases:												
			seed only (21) preirradiated												6 months

Fontanesi 1993 [118]	26 temporary	60Gy	Astrocytomas III (9)												20 months
		40-45cGy/h 7-25mCi	Glioblastomas (17) Additional pRT (66Gy@2x1.1Gy/d)												13 months
															

Voges 1993 [82]	1 temporary	40Gy	Ependymomas (1)								100%				
		3cGy/h 21.4mCi													

Kreth 1993 [104]	134 temporary	50-60Gy	Astrocytomas II (251)								64%				
	190 permanent	10cGy/h	Oligoastrocytomas II (44)								80%				
			Oligodendrogliomas II (29)								58%				

Sneed 1994 [136]	34 temporary	42-66Gy	Glioblastomas (34)			91%		40%	18%		9%				21 months
		30-60cGy/h	Additional pRT (56-60Gy)												

Wen 1994 [137]	56 temporary	50Gy	Glioblastomas (56)			83%		34%	27%	16%					18 months
		30-60cGy/h 20-50mCi	Additional pRT (59.4Gy @1.8Gy/d or 40Gy@2x2Gy/d)												

Bernstein 1994 [79]	46 temporary	70Gy	Recurrent tumours:												11 months
		68cGy/h	Astrocytomas II (2)												
		293mCi	Astrocytomas III (12)												
			Glioblastomas (32)												
			preirradiated (50-60Gy)												

Ryken 1994 [126]	15 temporary	60Gy or 80-300Gy	Recurrent tumours:												6 months
	5 permanent	40cGy/h	Astrocytomas III (9)												
			Glioblastomas (11)												
			Preirradiated (60Gy)												

Hitchon 1994 [125]	26		Astrocytomas III or Glioblastomas (26)												18 months

Fernandez 1994 [116]	73 permanent	100-120Gy	Astrocytomas III or Glioblastomas (73)			82%									> 31 months
		4-7cGy/h	Additional pRT (50-60Gy) (16% before, 68% concurrent, 15% not)												> 23 months

Kitchen 1994 [144]	23 temporary	50Gy	Recurrent astrocytomas III or glioblastomas (23)												6 months
		40-230mCi	Preirradiated (50-60Gy)												

Gaspar 1994 [119]	72 permanent	100Gy	Astrocytomas III (45)			80%		70%	62%						
		5cGy/h	Glioblastomas (27) Additional pRT (50-60Gy) (62% before, 24% after, 14% not)			80%		55%	40%						

Scerrati 1994 [105]	32 iridium-192	43Gy or 90Gy	Astrocytomas I (2)								83%	57%	39%		112 months
	4 iodine-125 14 temporary 22 permanent	32cGy/h or 42cGy/h	Astrocytomas II (23) Oligodendrogliomas (11) Additional pRT (30-54Gy)												

Zamorano 1995 [123]	72 permanent	5cGy/h	Astrocytomas III (45)			84%		75%	65%						
			Glioblastomas (27) Additional pRT (50-60Gy) (24% before, 63% after, 14% not)			80%		53%							26 months

Chamberlain 1995 [185]		45-51Gy	Recurrent tumours:												
	16 temporary	50cGy/h	Astrocytomas III (3)												9 months
			Oligodendrogliomas III (1)												
			Glioblastomas (11)												
			Ependymomas (1)												
			Preirradiated (54-60Gy)												

Kreth 1995 [68]	194 temporary 261 permanent	60-100Gy	Astrocytomas I (97)								85%		83%		
		< 10cGy/h	Astrocytomas II (250)								61%		51%		
			Oligoastrocytomas (60)								49%				58 months
			Oligodendrogliomas (27)								50%				61 months
			Gemistocytic gliomas (21)								32%				37 months

Fernandez 1995 [117]	58 permanent	102Gy	Astrocytomas III (40)												> 31 months
		4-7cGy/h	Glioblastomas (18)												> 23 months
		0.1-32mCi	Additional pRT (50-60Gy) (17% before, 66% after, 17% not)												

Sneed 1995 [141]	159 temporary	36-67Gy	Glioblastomas (159)			85%		36%	20%						19 months
		30-70cGy/h	Additional pRT (40-77Gy)												

Ostertag 1995 [150]	93 temporary	60-100Gy 10cGy/h	New metastases:												
			Seed + pRT (40Gy) (38)												17 months
			seed only (34)												15 months
			Recurrent Metastases: Preirradiated (21)												6 months

Halligan 1996 [128]	22 permanent	150-300Gy	Recurrent tumours:			57%									15 months
		11cGy/h	Astrocytomas III (4)												
		39mCi	Glioblastomas (18) Preirradiated (54-65Gy)			59%									15 months

Kreth 1997 [99]	197 temporary & permanent	45-100Gy or	Astrocytomas II (153)								60%				
		50-120Gy	Oligoastrocytomas II (44)												
		3-18cGy/h	Additional pRT (47)												17 months

Sneed 1997 [12]	permanent	60-100Gy	Astrocytomas I								85%		83%		
		5-10cGy/h	Astrocytomas II								61%		51%		

Sneed 1997 [12]	temporary	60Gy	New high-grade gliomas												18-19 months
		40-60cGy/h	Recurrent high-grade gliomas												12-13 months

Schulder 1997 [151]	13 permanent	43-132Gy	Metastases (13)												8 months
		0.26-0.79mCi	Additional pRT (30-75Gy)												

Chamberlain 1997 [138]	15 temporary	50Gy 50cGy/h	Recurrent glioblastomas (15)												10 months

Laperriere 1998 [24]	63 temporary	57-68Gy	Malignant astrocytomas (140)												
		21-125cGy/h	Additional pRT (50Gy) (63)												16 months
			only pRT (50Gy) (69)												13 months

Gaspar 1999 [53]	59 permanent	100Gy	Recurrent tumours:												
		5cGy/h	Astrocytomas III (22)			76%		55%	32%						16 months
			Glioblastomas (37)			44%		13%							24 months
			preirradiated (50-66Gy)												11 months
															
															

Videtic 1999, 2001 [121,122]	75 permanent	100Gy	Astrocytomas III (22)					58%			40%				40 months
		5cGy/h	Glioblastomas (53) Additional pRT (50-60Gy)					40-42%			18%				16-17 months

Suplica 1999 [142]	38 permanent	100-500Gy	Recurrent glioblastomas (38)												12 months
		0.67mCi	Preirradiated (59-72Gy)												

Patel 2000 [140]	40 permanent	120-160Gy 0.58-0.86mCi	Recurrent glioblastomas (40) preirradiated (60Gy)	72%											11 months

Koot 2000 [139] comment [196]	33 temporary 12 permanent	50-80Gy 3-5cGy/h 5-40mCi	Glioblastomas (45) Additional pRT (10-30Gy)												13 months

Rostomily 2001 [197]	6 permanent	160-218Gy	Recurrent paediatric tumours:												22 months
		10cGy/h 23-76mCi	Primitive neuroectodermal tumours (2)												
			Medulloblastomas (1)												
			Ependymomas (1)												
			Xanthoastrocytomas (1)												
			Glioblastomas (1) preirradiated (3 craniospinal, 2 WBRT)												

Keole 2001 [132]	26 permanent	83-100Gy	Glioblastomas (26) Additional pRT (57-61Gy)												23 months

Huang 2002 [153]	28 permanent	200-500Gy	Metastases:												11 months
		15-69mCi	New (16)												14 months
			Recurrent (12)												5 months

Zamorano 2003 [124]	60 permanent	100Gy	Astrocytomas III (57)			87%			60%		50%		46%		57 months
		3-7cGy/h	Oligodendrogliomas III (3) Additional pRT (50-60Gy) (78% before, 22% not)												

Larson 2004 [133]	35 permanent	150-500Gy 7-24cGy/h 56mCi	Recurrent glioblastomas (35) Preirradiated (59-72Gy)												12 months

Mehrkens 2004 [101]	55 temporary & permanent	60-100Gy 3-10cGy/h	Astrocytomas II (46) Oligoastrocytomas II (9)								55%		28%		68 months

Kreth 2006 [67]	136 temporary	100Gy	Astrocytomas II (187)								56%		37%	26%	
	103 permanent	60Gy 8cGy/h	Oligoastrocytomas II (52)												

Herrera 2007 [94]	12 temporary	52-80Gy	Paediatric astrocytomas I (12)					83%							
		4-20cGy/h 11-41mCi													

Julow 2007 [98]	43 temporary	40-200Gy	Astrocytomas I/II (27)			93%			61%		40%				36 months
		1-83cGy/h	Astrocytomas III (10)			48%			19%		0%				16 months
		12-155mCi	Glioblastomas (6)			17%			0%		0%				8 months
			New tumours (11)												
			Recurrent tumours (32)												

Dagnew 2007 [155]	26 permanent	120-200Gy	Metastases (26)			72%		46%							18 months

Chen 2007 [130]	18 permanent	200-600Gy	Glioblastomas (18)												26 months
		21-95mCi	Additional pRT (60Gy@ 2x1Gy/d)												

Schnell 2008 [102]	31 temporary	54Gy	New astrocytomas II (18)								93%				
		10cGy/h	Recurrent astrocytomas II (13)												

Darakchiev 2008 [131]	34 permanent	120Gy 27mCi	Recurrent glioblastomas (34)	82%		66%	37%	23%							16 months

Huang 2009 [156]	40 permanent	200-700Gy	New metastases (19)			47%		21%							12 months
		10-69mCi	Recurrent metastases (21)			33%		14%							7 months

Petr 2009 [157]	72 permanent	150Gy 4-40mCi	Metastases (72)			55%		27%							14 months

Schwarz 2009 [120]	71 temporary	20-60Gy	Astrocytomas I (4)												
		10-82mCi	Astrocytomas II (30)												41 months
			Astrocytomas III (11)												12 months
			Glioblastomas (13)												4 months
			Medulloblastomas (4)												
			Oligodendrogliomas (3)												
			Pinealis tumour (1)												
			Metastases (5)												
			Preirradiated (18-80Gy)												22 months

Ruge 2011 [149]	77 temporary	50Gy 6cGy/h	Metastases (77)												8 months

Korinthenberg 2011 [198]	94 temporary	60Gy	Paediatric tumours:								97%		92%		
		10cGy/h	Astrocytomas I (53)												
		10mCi	Astrocytomas II (26)												
			Oligoastrocytomas II (4)												
			Oligodendrogliomas II (5)												
			Ependymoma II (1)												
			Not classified (5)												

El Majdoub 2011 [183]	11 temporary	32-50Gy	Recurrent Medulloblastomas (12)			67%		33%							18 months
	1 permanent	2-40mCi	Preirradiated (24-56Gy)												

Ruge 2011 [154]	90 temporary	50Gy 3-6cGy/h	Metastases (90)												9 months

Suchorska 2011 [106]	95 temporary	50-65Gy	Astrocytomas II (69)												238 months
		3cGy/h	Oligoastrocytomas II (14)												
		12mCi	Oligodendrogliomas II (12)												

Ruge 2011 [199]	147 temporary	65Gy	Children:												
		1-31mCi	Astrocytomas I (101)			100%		98%			93%		82%		
			Astrocytomas II (34)								96%		85%		
			Other (12)								85%		77%		

Ruge 2011 [163]		50Gy	Metastases												15 months

#### Brachytherapy of pilocytic astrocytomas WHO grade I

Pilocytic astrocytomas frequently involve paediatric patients. Open tumour resection remains to be the gold standard, with favourable survival rates, provided complete tumour removal has been achieved. Unfortunately, these tumours are often found in highly eloquent locations such as the chiasmatic/hypothalamic area, the thalamus, the tractus opticus etc., rendering a complete resection impossible for a considerable number of patients. Poor physical, cognitive, and psychosocial outcome scores have been reported after long-term follow-up evaluation in particular for those patients with deep-seated tumour locations (diencephalon, optic chiasm, etc.). In here, an unfavourable outcome was at least partly attributed to side effects of surgical treatment [90-92].

In 1980, Mundinger et al. reported the first survival results of 55 young patients with diencephalic pilocytic astrocytomas treated with iodine-125 or iridium-192 interstitial brachytherapy. In this patient population 3- and 5-year survival rates of 72% and 52% have been achieved [4]. In paediatric patients the 1-, 3- and 5-year survival rates were slightly better for permanent iridium-192 (88%, 70% and 55%) than for iodine-125 implants (86%, 58% and 22%). Both nuclides proved to be more effective than biopsy or partial resection only [93]. Others report a 2-year survival rate of 83% for paediatric patients and temporary iodine-125 implants [94].

Another retrospective analysis revealed 1- and 3-year survival rates of 94% and 73% for newly diagnosed (treated in combination with percutaneous radiotherapy) and of 68% and 30% for recurrent (treated with brachytherapy alone) low grade gliomas [25].

The largest series with 106 pilocytic astrocytoma patients was published by Ostertag and Kreth. Patients were either treated with permanent or temporary implants; a 5-year survival rate of 77% was obtained and radiation toxicity was mostly observed in patients treated with permanent implants [95]. 5- and 10-year survival rates of up to 85% and 83% have been reported by another publication [68].

Of note, treatment of a subpopulation of 45 highly eloquent hypothalamic tumours was associated with low risk and provided similar clinical outcome rates. Peraud and co-workers report on clinical outcome after temporary iodine-125 implantation (54Gy, 8cGy/h, < 20mCi) for complex located WHO grade I and II gliomas (including 4 mesencephalic/pontine, 2 thalamic/pineal and 2 mesencephalic/pontine tumour locations) [72]. A complete response after brachytherapy was seen in four patients, and a partial response in seven patients. None of the patients exhibited tumour progression within a median follow-up of 31.5 months, and no radiogenic complications occurred. Of note, functional outcome scores were favourable with significant improvement of pre-existing hemiparesis in 3/4 patients, improvement of endocrine deficits in one half of patients, and improvement of visual functioning in 1/3 patients. Other retrospective data are in line with these findings [94]. Of note, long term side effects of brachytherapy seem to be rare in the paediatric subpopulation [96].

To summarize, studies on brachytherapy in pilocytic astrocytomas WHO grade I are rare and retrospective. Therefore, no clear conclusion can be drawn concerning indications for brachytherapy for these tumours. However, stereotactic iodine-125 brachytherapy has been shown to be a safe, minimally invasive, and an effective first-line treatment option for selected patients with highly eloquent tumour locations, bearing a high risk for open tumour resection. As pilocytic astrocytomas are mostly slow growing, sharply marginated tumours in the midline and highly eloquent areas, brachytherapy should be considered an attractive option.

#### Brachytherapy of gliomas WHO grade II-IV

Many more patients with gliomas WHO grade II-IV have been treated with iodine-125 implants. However, the place of interstitial brachytherapy remains poorly defined. Moreover, the effects of an oligodendroglial differentiation, as well as recently discovered molecular genetic markers (such as LOH1p/19q, TP53 mutations, MGMT promoter methylation and IDH1 mutations), on the efficiency of interstitial brachytherapy, have not been evaluated yet [16].

#### Brachytherapy of gliomas WHO grade II

Results of brachytherapy for low grade gliomas WHO grade II - as first line treatment option - have been already summarized before [97]. Most data come from retrospective studies: 1-, 3- and 5-year survival rates in the range of 79-94%, 36-73% and 21-65% for primary tumours and 1- and 3-year survival rates of around 68% and 30% for recurrent tumours have been reported [4,12,25,67,68,93,95,98-101].

The response rates of astrocytomas WHO grade II for iodine-125 brachytherapy included 7.5-25.8% complete responses, 13.8-29.0% partial responses, and 45.2-61.1% stable diseases. The rate of non-responders ranges between 0% and 17.6% [67,102].

One of the largest long-term analysis (median follow-up: 10.3 years) reports on interstitial brachytherapy in 239 patients with progressive eloquently located circumscribed, supratentorial WHO grade II gliomas [67]. In 103 patients permanent implants were used, since 1985 temporary implants have been preferred. Five-, 10-, and 15- year progression free survival was 45%, 21%, and 14%, respectively. The corresponding survival rates were 51%, 32%, and 22%, respectively. Of note, no levelling off of the Kaplan Meier curves was observed and patients experienced tumour progression even 10 years after treatment. Complete response was seen in 18 patients, partial response in 33 patients, tumour control in 146 patients, and unrestrained tumour progression in 42 patients (non-responder group). Transient radiogenic complications occurred in 19 patients.

Schnell et al. found a 5-year survival rate as high as 93% in patients with low activity temporary iodine-125 implants (54Gy, 10cGy/h) [102].

Malignant transformation of astrocytomas II was seen in 33% of the patients after 5 years, in 54% after 10 years and in 67% after 15 years [67], but was not different to open tumour resection alone [16].

In a retrospective analysis, Warnke and co-workers observed a significant reduction of seizure incidence and increase of benzodiazepine receptor density (as demonstrated by single photon emission computed tomography in a subset of 20 patients), after brachytherapy of 80 patients with temporal WHO grade II astrocytomas [103]. Of note, 79% of patients became seizure free after six months.

For astrocytomas grade II, a significantly better outcome with less radiation toxicity was reached with temporary seeds than with permanent ones, which are associated with an increased risk for prolonged oedema [95,100,101,104]. Target volume and radiation dose showed a direct correlation with the risk of radionecrosis with critical values being 35 cm^3 ^and 100Gy for permanent and 50Gy at 42cGy/h for temporary implants [105]. Age, KPS, midline shift, tumour volume and enhancement on CT were predictors for outcome [68,95,99-101].

A combination of microsurgery and stereotactic brachytherapy for tumour remnants is feasible and might be an alternative treatment strategy, for example, for large lesions with significant proportions of eloquent tumour parts to avoid neurological deficits [16]. Brachytherapy to treat progressive or recurrent tumours after resection achieved a tumour response rate of 35.9% and a control rate of 97.3% after 24 months; median progression free survival was 53 months [106].

All in all, even though stereotactic brachytherapy has been used for low-grade gliomas for many years, its place within the multimodality treatment concept is still debated [16]. Small (less than 4 cm in diameter), circumscribed lesions in highly eloquent or deep-seated tumour locations might be good candidates for interstitial brachytherapy, especially in cases harbouring an increased risk for open tumour resection. Moreover, radiobiology, tumour shape and the option of percutaneous radiotherapy for recurrence, favour an increased application of brachytherapy in astrocytomas grade II.

#### Brachytherapy of anaplastic gliomas WHO grade III

For newly diagnosed anaplastic astrocytomas WHO grade III, standard therapy consists of radiation therapy with/-out surgical resection, whenever safely possible. Alternatively, first line chemotherapy might be considered (NOA-04 study). However, interstitial brachytherapy might also be a local treatment option, especially for those patients that are not clear surgical cases.

For newly diagnosed anaplastic astrocytomas, the combination of interstitial brachytherapy with percutaneous radiotherapy has been shown to be associated with 1-, 2-, 3- and 5-year survival rates of 33-87%, 36-75%, 17-66% and 0-50%. Median survival is also highly variable and ranges between 8-57 months. Patients treated with seeds for recurrent anaplastic astrocytomas had 1-, 2- and 3-year survival rates of 49-81%, 46-55% and 24-40% and a median survival of 9-35 months after implantation [4,23,25,44,45,53,81,93,95,98,107-124].

Two studies found an enhanced median survival in patients treated with percutaneous radiotherapy in combination with temporary iodine-125 implants, compared to external irradiation alone [24,125]. While in the NCOG 6G-82-2 study, the brachytherapy boost had no additional advantage [23].

Gutin et al. compared survival after iodine-125 brachytherapy for recurrent anaplastic astrocytomas to that of a historical chemotherapy group and obtained better survival data for the brachytherapy group [110].

Factors associated with improved survival were age ≤ 45-50 years, KPS ≥ 80-90, superficial location, decreasing volume of implanted tumour, chemotherapy at recurrence and reoperation at the original site in case of tumour recurrence [24,117,124,126].

Different schemes have been used for primary therapy. On the one hand, 50-60Gy of temporary implants have been combined with 50-60Gy percutaneous radiotherapy [24,81,107]. On the other hand, 100Gy of permanent implants have been added to 50-60Gy external beam radiotherapy [124]. Sometimes seeds had been implanted before percutaneous radiotherapy had been started [108].

There was as well a lot of variation in therapy for recurrent, pre-irradiated astrocytomas III. Doses of 50-150Gy have been employed with temporary implants [44,45,107,111,127]; 100-300Gy with permanent [53,128].

To summarize, multiple, even several randomized, studies were performed to analyze the value of brachytherapy in the primary treatment of astrocytomas grade III, but failed to prove any clear beneficial efficacy for this tumour entity. Therefore, standard therapy is radiotherapy or chemotherapy according to the results of the NOA-04 study [129]. However, for recurrent, pre-irradiated tumours, seeds might be a valuable option.

#### Brachytherapy of glioblastomas WHO grade IV

Interstitial brachytherapy has been repeatedly suggested for malignant gliomas WHO grade IV at primary diagnosis, as well as during the course of the disease. For primary glioblastomas, 1-, 2-, 3- and 5-year survival rates of 80-91%, 19-57%, 14-40% and 9-18% and a median survival of 13-26 months could be achieved. 1-, 2- and 3-year survival rates for recurrent tumours were 44-66%, 13-26% and 8-15%, and median survival 6-17 months [4,23,25,44,45,53,81,95,108-119,121-123,128,130-142].

Several retrospective studies showed a better survival for patients with primary glioblastomas treated with a temporary or permanent brachytherapy boost, in addition to parallel or sequential external beam irradiation, instead of percutaneous radiotherapy alone (e.g. 1-, 2-, 3- and 4-year survival rates have been 40%, 12.5%, 9% and 5% compared to 83-87%, 34-57%, 27% and 16%; median survival 11 months vs. 18 months) [134,137]. There was no survival difference for iridium-192 and iodine-125 [139], or brachytherapy and radiosurgery boost [132], but permanent implants showed improved survival compared to temporary [115].

Percutaneous and interstitial irradiation in newly diagnosed paediatric malignant gliomas have been complemented by high-dose chemotherapy and autologous bone marrow rescue, but was not associated with better outcome scores than for conventional regimens [143].

For recurrent glioblastomas, tumour resection plus permanent seed implantation was compared to biopsy plus temporary seed implantation, and revealed similar median survival rates [133].

Subgroup analyses of the results for primary treatment of anaplastic astrocytomas and glioblastomas with percutaneous radiotherapy and permanent brachytherapy boost, divided into the RTOG recursive partitioning analysis (RPA) classes, have shown the following results: the 2-year survival rate and the median survival for RPA classes I/II were 68% and 37 months, for class III 74% and 28-31 months, for class IV 32-34% and 16 months and for classes V/VI 29% and 11 months. Iodine-125 has improved the survival most demonstrably in poorer prognostic classes so that a selection bias does not account for the survival benefit [121,122].

Age has an important effect on survival with a median survival of 109 weeks for patients aged 30-40 years, 96 weeks for 40-50 years, 77 weeks for 50-60 years and 76 weeks for 60 years and older [141]. Other factors affecting outcome were KPS, superficial lesion, gross total resection, reoperation, residual tumour volume, number of seeds, seed activity, volume implanted and dose [25,117,126,140,142,144].

A recurrence pattern analysis of patients treated in the NCOG 6G-82-2 trial revealed that 77% were local (within 2 cm from seed), 14% separate, 5% subependymal and 5% systemic [136]. Others found 35% local, 65% marginal and 28% distant relapse [137] or 70% local, 18% distant and 12% both [128].

Many different dose combinations of percutaneous radiotherapy and iodine-125 brachytherapy have been given in the primary therapy setting; 45Gy whole brain radiotherapy and 100-280Gy (15-30cGy/h) brachytherapy boost [47], 59.4Gy (1.8Gy, 5×/week) or 40Gy (2Gy, 2×/day) and a high activity brachytherapy boost of 50Gy [137], but most percutaneous and temporary seed dose were 60Gy [139,141]. Sometimes radiotherapy was combined with chemotherapy regimens [109,118,125,138] or hyperthermia [145], or was given after seed implantation [108].

For pre-irradiated, recurrent glioblastomas, seeds have been implanted only without further percutaneous radiotherapy. 100-500Gy of permanent seed dose have been chosen [140,142] or 50-120Gy of temporary [44,110,111].

All in all, multiple studies for newly diagnosed glioblastomas have been performed, but brachytherapy does not play a role in this setting nowadays. Standard therapy is surgery and radiotherapy, plus concomitant and adjuvant temozolomide [146]. In the recurrent setting, brachytherapy might be an option as interstitial irradiation can still be employed in cases in which tolerance of the healthy brain tissue has been reached because of previous external irradiation [4]. Interstitial irradiation offers excellent palliation in a majority of patients with a significant amount of long-term survivors for recurrent gliomas [147].

#### Brachytherapy of brain metastases

Besides primary brain tumours, interstitial brachytherapy should be considered for brain metastases [148,149]. Some accept a maximum diameter of 4 cm [150], others implant 3-6 cm metastases that are beyond the size limit of radiosurgery [151]. The advantages of treating brain metastases with brachytherapy are a minimally invasive procedure, a minimal damage to normal brain tissue, no supplementary risk by anaesthesia, no interference with other therapeutic modalities, low stress for the patient, a short hospitalisation time (2-3 days) and low costs. The reasons why brain metastases are ideal for interstitial irradiation are the frequent spherical shape, the mostly relatively small size, the normal brain parenchyma being displaced outside the potential target volume and the minimally invasive growth [152].

For metastases, local control rates of 73-95% have been reported [153,154].

Median survival after implantation varies from 4 to 18 months, and 12- and 18-months survival rates from 33 to 72% and from 25 to 33% [104,115,145,149-151,153,155-161].

Kreth et al. compared the median survival after iodine-125 brachytherapy (60Gy, 10cGy/h, temporary), percutaneous radiotherapy (40Gy, whole brain radiotherapy) and a combination of both, for newly diagnosed brain metastases, and observed median survival times of 12-15 months, 8 months and 17 months, respectively. For recurrent metastases, survival times were around 6 months [104,150]. Results for singular brain metastases are comparable with stereotactic radiosurgery [149].

For metastases, good prognostic factors are KPS ≥ 70, solitary metastasis, no extracerebral metastases, long time interval between primary diagnosis and diagnosis of metastases [104,150,162].

A potential risk is growth of tumour cells by inoculation along the catheter pathway, which was seen by Ruge et al. in 6 out of 90 patients [154].

All in all, iodine-125 brachytherapy for metastases has become more popular again in recent years due to its radiobiological advantages and as it can be employed even after extensive percutaneous pre-irradiation [120], additionally biopsies are possible in the same session [154,163].

#### Brachytherapy of pineal parenchymal tumours

Iodine-125 seeds (50-60Gy, 9-12cGy/h) have been used for recurrent pineoblastomas [164]. A study of 18 patients (6-68 years) treated with 40-65Gy of permanent (11) or temporary (7) implants for primary pineal tumours or in a salvage situation has been published. 5 year overall actuarial survival rates of 100% for pineocytomas and of 78% for high-grade tumours have been reported. Complete remission rate was 72% and partial remission rate 28%. Maarouf et al. concluded that iodine-125 seeds are efficient and safe and an attractive alternative to microsurgery [165]. All in all, iodine-125 brachytherapy treatment with a low dose rate seems to be an attractive, minimally invasive treatment option of these rare tumour entities of the pineal region.

#### Various indications of brachytherapy

One group implanted petroclival meningiomas [26]. Treatment of meningiomas of the skull base and other localisations with permanent iodine-125 implants results in a complete response rate of 73-82% and a partial response rate of 18-27%, without early, and with rare late complications [166-170]. The treatment of parasellar-clival and globoid meningiomas with permanent implants showed a slow reduction in volume with no mortality [171]. All these results are only of historical interest as seeds do not play a role in modern treatment of meningiomas.

Case reports present successful treatments of an example of bilateral blindness secondary to a recurrent, preirradiated hemangiopericytoma of the pituitary fossa [172], or of radiation-induced neoplasms [173]. Even a clival chordoma [174] and other skull base tumours [175,176] have been treated with an implantation of seeds, as well as locations that are a challenge for high-precision percutaneous radiotherapy [177,178]. A case of Rosai-Dorfman disease of the central nervous system has been cured with brachytherapy [179], as well as child optic pathway gliomas [180]. Craniopharyngiomas have been implanted without recurrences [181,182] and recurrent medulloblastomas have been treated with iodine-125 brachytherapy [183]. Sometimes iodine-125 implants may have a favourable long-lasting effect upon medically refractory seizures [184].

### Side effects and complications

The following side effects and complications have been described in the literature: intracranial pressure and tumour expansion due to necrosis, severe oedema, cerebral artery occlusion, intracranial haemorrhage and subdural bleeding, headaches, nausea, vomiting, seizures, increased neurological deficits, abscesses, aseptic and septic meningitis, wound infections and dehiscence, delayed wound healing, scalp infections, cerebrospinal fluid leakage, long-term steroid dependency, progressive dementia, psychotic symptoms, facial pain, pulmonary embolism and others [12,13,22,24,45-47,70,81,101,107,110,114,134,137,138,155,185]. Two cases of devastating strokes due to intracranial arterial occlusion have been reported [43]. Tacke et al. found in 6 out of 13 children with low-grade hypothalamic gliomas signs of vasculopathy in magnetic resonance imaging, but only one of them revealed symptoms of intermittent cerebral ischemia [186].

No toxicity was reported in 92%, severe acute toxicity in 6%, life threatening in 1% and fatal in < 1% [25]. All in all, the operative morbidity seems to be low (1.2-21.7%) [67,79,95,107,150,155]. In patients with gliomas grade II, a combination of surgery plus iodine-125 brachytherapy had a morbidity of 27.8%, seed implantation alone of 6.4% [102]. The perioperative mortality rate is max. 0.8-2.6% [4,67,68].

Radiation toxicity (oedema) has been reported mostly among patients treated with permanent implants [101,104]. It occurs in 1.7-7.5% [68,95,96,101,104], although for tumours greater than 3.5 cm in diameter, it occurs in up to 18% of cases [101]. Late radiation necrosis is a severe side effect which seems to occur in 40% of the low-dose-rate implantations and is correlated with total radiation dose, implanted activity and the velocity of tumour shrinkage. It can be avoided by the usage of temporary implants [85]. A detailed analysis of patients with grade I and grade II gliomas undergoing low-activity iodine-125 brachytherapy has revealed as risk factors for radiogenic complications the volume of intratumoural 200Gy isodose and rapid tumour shrinkage of ≥ 50% of the volume in six months. A 200Gy-isodose-volume of < 4.5ml leads to < 3% radiogenic complications with a steep increase of the risk beyond this limit. For temporary implants with a reference dose of 60Gy, the risk for complications is < 3% if the treatment volume is ≤ 23 ml. Further factors affecting the toxicity rate examined are the overall treatment volume, the volume of the 60Gy isodose outside the target volume, reimplantation, the reference dose, the number of implanted seeds and a lobar tumour location [96]. Other authors identified a target volume > 35ml and a permanent seed dose > 100Gy, or a temporary > 50Gy, as risk factors of complications [105]. Evaluation of a brain necrosis after permanent seed implantation showed the necrotic area within the 100Gy isodose and damage to the blood-brain barrier within the 50Gy isodose [187]. A long term analysis of Kreth et al. revealed a risk for radiogenic complications of about 9% for relatively small grade II gliomas (diameter < 4 cm) and a steep increase of complications (about 25%) for those harbouring larger tumours (≥ 4 cm). The increase of radiogenic complication rate beyond a critical threshold can be explained by the tissue effects of brachytherapy, such as the increase of capillary permeability in the vicinity of the high-dose zone and the exponential increase of the damaged capillary surface area product with the square of the radius of the high-dose zone [16]. The risk of low-dose rate brachytherapy of low-grade gliomas should not be confused with the high frequency of complications (40-50%) after high-dose rate treatment of malignant gliomas. It remains difficult to elucidate, in which extent, additional external beam radiation, the often relatively large size of the treatment volume, and the applied high dose rate (in the range of 35-50cGy/h), have contributed to these high complication rates. Reoperation has been shown to be required in 0-72% because of treatment induced space occupying lesions, which occurred particularly after high dose rate brachytherapy 3-183 weeks after implantation [23,25,44-46,53,79,81,95,101,104,105,107,109-114,116-119,123,124,127,130,132,133,135-139,144,150,155,185,188,189]. No correlation between implanted volume and need for reoperation has been found [137]. However, there might be a correlation for the combination of percutaneous radiotherapy plus brachytherapy (64%), compared to percutaneous radiotherapy only (15%) [137], others found similar rates of around 30% for both groups [24]. Histological findings (necrosis, tumour) at reoperation are not prognostic factors [190].

Especially permanent seeds and periventricular location, carry the risk of seed migration [133,191] but temporary seeds also sometimes need repositioning [46,120].

Percutaneous catheter derived brachytherapy may be associated with an increased incidence of extraneural metastatic gliomas of the scalp, skull, cervical nodes, etc. [192].

#### Quality of life

The quality of life in the majority of long-term survivors appears to be quite satisfactory [23,44,45,193]. Comparing permanent and temporary implants, the KPS remained stable or improved in 85% and 58%. The neurological status emended in 87% and 67% [115]. In another study the neurological function has improved in 30%, has shown no change in 43% and has declined in 27% [95]. The KPS mostly improved in patients with astrocytomas II [104].

#### Summary and proposals for reduced side effects

In summary, to reduce the risk for side effects, temporary implants, dose rates around 10cGy/h and activities < 20mCi should be preferred, high dose zones > 150Gy should not be located within normal tissue and next to vessels, and volumes > 4 cm should not be implanted. If these guidelines are followed, then side effects are very rare [16].

## Conclusions

Iodine-125 brachytherapy might be indicated in highly selected patients with recurrent brain tumours as a form of re-irradiation, low-grade gliomas, brain metastases with stable systemic disease and some other less common diagnoses, as it is a way to deliver an additional dose of localized radiation to malignant brain tumours while limiting radiation to the surrounding normal brain tissue [13]. However, there is no strong randomized evidence that would justify the change of guidelines for any brain tumour entity. The side effects are rather rare. During follow-up, attention has to be paid to some special diagnostic features. In that case, iodine-125 seed implantation in brain tumours may lead to good survival rates and a decent quality of life.

## Competing interests

The authors declare that they have no competing interests.

## Authors' contributions

SBS designed the protocol, conducted data evaluation and wrote the article. NT also wrote the article. KN, MN, JCT, CB and FWK performed a critical review of the manuscript. CB and FWK also designed the protocol. All authors read and approved the final manuscript.
